# Policy Horses Still Running Around Healthcare Courses: A Response to Recent Commentaries

**DOI:** 10.34172/ijhpm.2024.8454

**Published:** 2024-03-04

**Authors:** Martin Powell, Russell Mannion

**Affiliations:** Health Services Management Centre, University of Birmingham, Birmingham, UK.

 We wish to begin by expressing our appreciation to the authors of the commentaries for their constructive engagement with our editorial – Modelling the Health Policy Process: One Size Fits All or Horses for Courses?^1^ We apologise that it is impossible in the space provided to respond to all of the individual points made by the seven commentaries containing over 100 references. Each commentary adds to the scholarly conversation surrounding health policy processes and we are pleased to have opened up a debate where it seems that the authors have views that are similar in some cases (where later commentators agree with earlier ones) and different in other cases. The “headline” responses to our main question of whether we should analyse health policy processes in the same way as other policy processes (a “One Size Fits All” approach) or use more health-specific explanatory models (“Horses for Courses”) seems to be the former for three or four responses, perhaps the latter for one response, and both for one or two responses.

 Each commentary engaged critically and imaginatively with the main ideas we presented, offering some degree of affirmation of our argument, but also taking the discussion in new directions. Some commentators are correct that we set out a narrow view of the policy process in a number of ways. In a short “Editorial” this was necessarily the case. Our simple method was “deductive” rather than “inductive,” with public policy theories taken (not unreasonably) from one well regarded text entitled “Theories of the Policy Process.” Flowing from these theories, we accept that we tended to focus more on, in the terms set out by the Health Policy Analysis Triangle, policy process rather than policy content, actors, and context. Similarly, we concede that we could have differentiated more clearly understanding policy-making by describing and explaining policy processes from understanding policy-making to use that knowledge to evaluate or seek to influence policy change. Finally, we accept that the ‘big three’ models of the policy process—Multiple Streams Analysis, Advocacy Coalition Framework, and Punctuated Equilibrium Theory—have been criticised for their limited unpacking of institutions.

 Responding to each contributor in a little more detail, Greer^2^ discusses three issues. First, there is some health policy parochialism, notably as seen in theories only found in health policy scholarship. Second, scholarly progress is possible through the simple application of well-known theories. Third, there is the question of whether theories of the health policy process are studying the right thing at all. However, his main point is that developments in some countries tend to be explained by politics over time and path dependency, which are dynamics that are difficult to capture in any “theory of the policy process.” He argues that policies and legacies can be more important than formal political institutions. In short, policy creates politics.

 Changing the pace, Cairney^3^ sets out two very pertinent questions of: what would health researchers be trying to do, and why when using policy theories; and what practical lessons can policy theories provide and not provide?

 In their contribution, Gilson and Walt^4^ ask three sets of questions – about theory use, the deepening of health policy process research and its substantive relevance. First, they correctly remind us about the importance of context. In particular they wonder if the existing body of public policy theory is fit-for-purpose in examining health policy processes in low- and middle-income countries (LMICs)? They support Rod Sheaff’s claim that a bricolage approach may be best in conducting such research, drawing from a range of theory, both from beyond and within the health policy terrain. Second, they argue that using theory is not the most important factor in deepening research about health policy processes. Third, they urge us to consider the relevance of generating understanding of health and other policy processes. Like Cairney, they consider what practical value theory has for those engaged in bringing about policy change. From this perspective, they see value in the continued and wide application of the health policy analysis triangle.

 Harris^5^ is correct in his analysis that our reliance on theories of the policy process from one text underemphasises institutions, and under-plays the dynamic role of structure and agency.

 Parkhurst^6^ supports the “universal” approach, as it is difficult to argue that health policy-making is inherently different from other social policy sectors, and making an argument for “health policy exceptionalism” appears unjustified. He is correct that our category of other “Health Policy Process” models is due to being applied to health cases, but not necessarily because they are developed for health specific issues. Put another way, these models were not discussed in the broad public policy text that we drew on. He notes that the most commonly cited work in policy sciences comes from the United States and Europe, but this may not be simply applicable to health reforms and policy-making in LMICs (cf Gilson and Walt^4^, Peckham,^7^ and Sheaff ^8^).

 Peckham^7^ argues that our analysis is based on a narrow range of examples with a specific focus on “models” AND “policy,” and “health.” He is correct that we did not engage with a much broader literature as this was not discussed in our material uncovered by our review. He points to a wider health policy literature, particularly analyses of health policy in LMIC, and to political and organisational, governance and systems theories. As above, we accept that this was necessarily the case in a Short Editorial. We are not sure that he is correct that “most analyses of policy rightly draw on multiple policy lenses,” or indeed that more analyses should draw on approaches that combine approaches within governance and systems analyses. We tend to agree with Greer that as simple models are sometimes not well applied, there may be even more problems with more complex models.

 Sheaff ^8^ favours a “bricolage” approach that leads to a further question of how to reveal the politics within the health policy process. He makes the interesting point of considering how the realist idea of a context-mechanism-outcome configuration relates to the models reviewed. He argues that a high-generality model of “one size fits all” policy processes including health needs to be qualified (not replaced) with additional explanations of how and why policy processes in particular polities are distinct special cases of that general model; and then how and why the health policy process is a still more special case within that: a “horses for courses” approach. This in turn shows that the health policy process depends on more than actors, “contents” and processes within the health system alone, but also introduce politics.

## Conclusion

 We feel that the valuable insights provided by this collection of commentaries have, as intended, stimulated debate for those interested in researching and analysing health policy processes. To return to the “headline” response to our main question, we agree with the majority of commentators that the “One Size Fits All” horse is still leading the “Horses for Courses” horse… albeit perhaps by a short head.

## Ethical issues

 Not applicable.

## Competing interests

 Authors declare that they have no competing interests.
